# Insights from molecular network analysis to docking of sterubin with potential targets

**DOI:** 10.6026/973206300191184

**Published:** 2023-12-31

**Authors:** Sittarthan Viswanathan, Kavimani Subramanian, Vimalavathini Ramesh, A. Hannah Rachel Vasanthi

**Affiliations:** 1 Department of Pharmacology, Mother Theresa Post Graduate & Research Institute of Health Sciences (Government of Puducherry Institution), Puducherry - 605006, India; 2 Department of Biotechnology, Pondicherry University - 605014

**Keywords:** Sterubin, Eriodicyton californicum, network analysis, molecular docking, Alzheimer's disease

## Abstract

The use of a flavonoid compound sterubin in drug discovery is gaining momentum. Hence, it is of interest to document the molecular network analysis to docking of sterubin with potential targets to glean insights. We identified 32 target genes and (or) gene products for sterubin using DAVID tools for GO, KEGG pathway enrichment analyses and the STRING database. Further, molecular docking analysis data of sterubin with these targets is documented for further consideration in broad-spectrum drug discovery.

## Background:

The medicinal properties of plants are mostly attributed to their secondary phytochemical metabolites. These natural products, which have evolved over millions of years, have a unique chemical diversity that results in immense biological activities and drug-like properties [1]. Secondary metabolites are further categorized into a number of groups, including glycosides, tannins, terpenoids, alkaloids and phenyl-propanoids and allied phenolic compounds, depending on their biosynthetic origins [2]. Natural polyphenols from plants are called flavonoids, which are naturally occurring compounds that are biosynthesized from phenylalanine, and are ubiquitous to green pigments in the plant kingdom [3]. Until now, more than 7,000 flavonoids have been reported from natural sources including medicinal plants, vegetables, fruits and wines [4]. They are grouped into a variety of sub-classes according to their chemical composition and the different types of substituents present in their aromatic rings, namely flavanones, flavonols, flavones, isoflavones, dihydroflavones, chalcones, anthocyanidins and catechins.

Natural O-methylated flavones, flavanones, and chalcones are the majority of them. Some of these compounds have also been found to apply beneficial physiological effects. Sterubin which as a potent antioxidant, free radical scavenger, and metal chelator, also presents anti-cholinesterase, anti-aging, neuroprotective and anti-inflammatory properties and neuro-trophic roles, ameliorating learning and memory, possessing potent antidepressant and anti-amyloidogenic effects, suppressing the activation of microglia, and mediating inflammatory processes in the central nervous system (CNS) [5].

Sterubin (7-O-Methyleriodicytol) is a flavanone compound from the leaves of Eriodicyton californium, Eriodicyton angustifolim (Yerba santa). It has a broad range of pharmacological properties such as high neuroprotective, anti-inflammatory, anti-oxidant, anti-amyloid and it is used to treat respiratory ailments such as cough, cold, asthma, bronchitis and age-related complications. Sterubin has been identified through old age-associated phenotypic screening [6]. Sterubin exhibits antioxidant activities by protection against oxytosis (oxidative glutamate toxicity) in HT22 cell line with an EC50 0.8 µM. Moreover, in a short-term model of AD the amyloid beta (Aβ) peptide injected into the cerebral ventricles, was able to prevent Aβ-induced decreases in short and long-term memory [7]. Therefore, it is of interest to document the network and molecular docking analysis data of sterubin with potential targets to glean insights.

## Methodology:

## PubChem database-based screening of chemical structures and ADMET Analyses:

PubChem is a free and open database containing important information about drug development and chemical biology research PubChem [8]. The chemical formula and SMILES of sterubin were found by entering the term "Sterubin" in the search box. ADMET analysis for sterubin was performed using the pkCSM web tool [9]. The procedure for sterubin gene prediction and analysis is shown in Table 1.

## Screening of possible target for sterubin using binding database:

A free online Binding DB database covers protein interactions with small drug-like compounds. It was connected to numerous databases, and these connections were used to extract further information regarding the targets. Using SMILES and the "homo sapiens" setting in Binding DB (https://www.bindingdb.org/bind/index.jsp) by selecting the "minimum needed interaction score" to "high confidence (0.700)" throughout the prediction phase, the target genes was evaluated in Binding DB [10].

## Protein-Protein interaction network construction and analysis:

STRING 11.0 is an online database that collects, assesses, and integrates information regarding protein-protein interactions from publicly available sources (http://string-db.org) [11]. It can enhance the existing data on protein-protein interactions with computational predictions. The STRING database contained 58 additional potential sterubin targets. The species was set to Homo sapiens, and the minimum interaction score was set to 0.7 to create a protein interaction network. For visual analysis, the findings were loaded into Cytoscape 3.7.2. The degree was calculated to identify core targets by the Network analyzer plugin (http://appss.cytoscape.org/apps/net-workanalyzer). A higher degree value node represented putative crucial targets of sterubin in the PPI network. The top 10 targets were selected according to the degree as core targets.

## Gene ontology and KEGG pathway enrichment analysis:

The biological process (BP), molecular function (MF), cell component (CC), and Kyoto Encyclopedia of Genes and Genomes (KEGG) pathway enrichment were analyzed using the Database for Annotation, Visualization, and Integrated Discovery (DAVID) (https://david.ncifcrf.gov/) to explicate the role of target proteins that interact with sterubin [12].

## Construction of sterubin-target-pathway network:

The top 20 pathways were evaluated using DAVID based on KEGG pathway enrichment analysis to reflect the relationship between sterubin-target-pathways network was constructed through Cytoscape 3.7.2 software.

## Molecular docking:

For the molecular docking, 10 target genes such as HSP90 AA 1, AKT-1, ESR-1, RELA, ESR-2, AR, APP, PPAR-δ, STAT1 and HSP90 AB 1 were selected by comparing the hub genes with the results provided by KEGG analysis pathways. The sterubin were docked with these potential targets. The structures of sterubin were retrieved from the PubChem database. The selected 3D structure of the ligands was retrieved from PubChem compound database in SDF format followed by conversion in the PDB format and optimization using Discovery Studio. The lower (more negative) the binding energy, the stronger the anticipated affinity for binding of the ligand against the target in molecular docking. Protein Data Bank was used to obtain the crystal structures of target genes HSP90 AA 1, AKT-1, ESR-1, RELA, ESR-2, AR, APP, PPAR-δ, STAT1 and HSP90 AB 1. Prior to docking analysis, prominent active site prediction of these selected targets was carried out by PDB Sum database. The active site region is given in Table 3. Molecular docking was carried out using Auto dock 4.2.1 software based on Lamarckian Genetic Algorithm; which combines energy evaluation through grids of affinity potential to find the suitable binding position for a ligand on a given protein. Grid maps were generated by Auto Grid program. Each grid was cantered at the crystal structure of the corresponding targets. The grid dimensions were 60 Å X 60 Å X 60 Å with points separated by 0.375 Å. For all ligands, random starting positions, random orientations, and torsions were used. The Docking parameters Number of Genetic Algorithm (GA) runs: 25, Population size: 150, Maximum number of evaluations: 2,500,000, Maximum number of generations: 27,000 were used for this study. The structure with the lowest binding free energy and the most cluster members was chosen for the optimum docking conformation.

## Results and Discussion:

**Figure 1:**
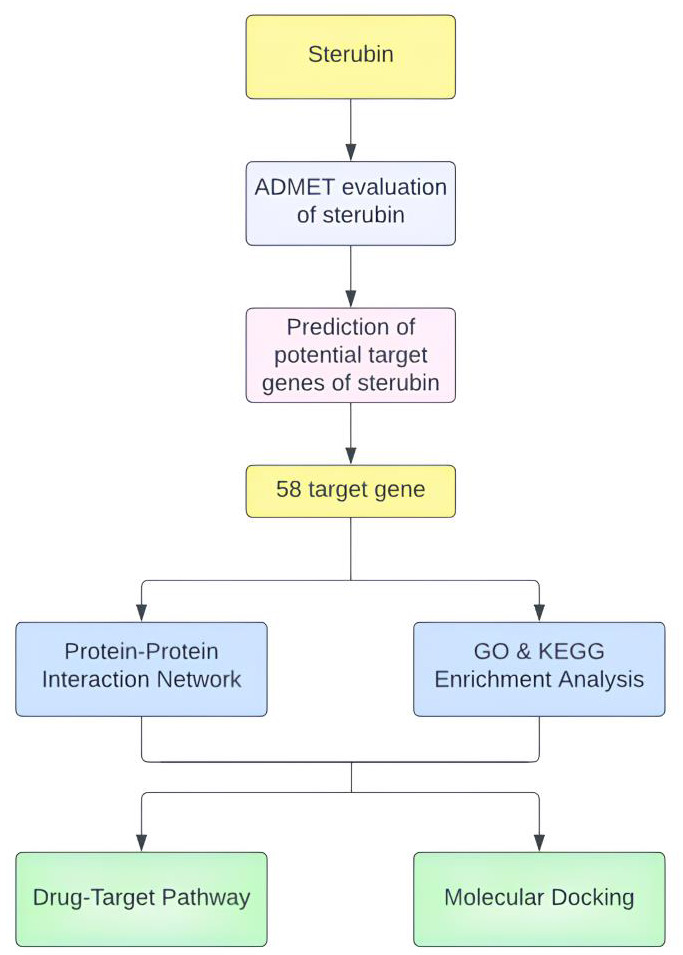
The overall work flow diagram

**Figure 2:**
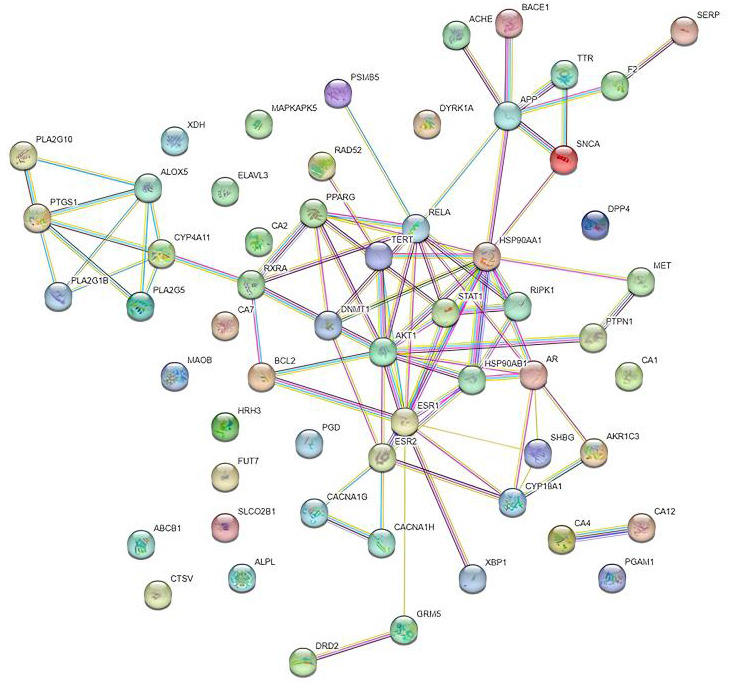
Protein interaction network for sterubin

**Figure 3:**
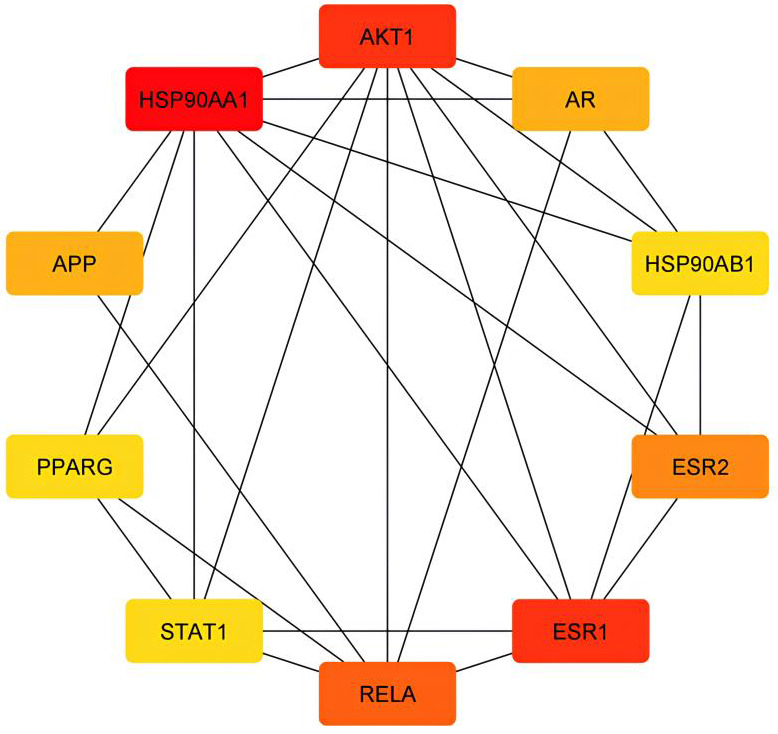
Top 10 degree of sterubin targets

**Figure 4:**
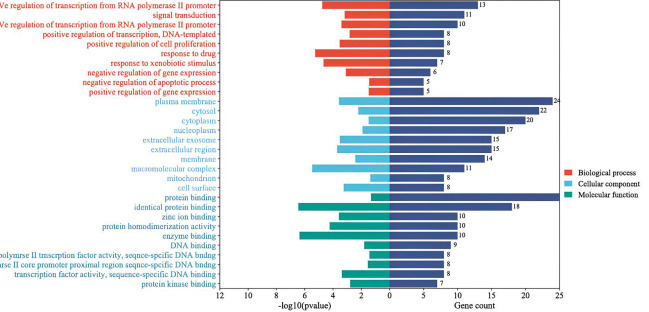
GO enrichment analysis of target genes. Top 10 selected according count of the gene of BP, CC & MF

The SIMLES and chemical formula of sterubin were retrieved from the PubChem database (Figure 1). The ADMET analysis of sterubin was conducted using the online tool pkCSM, and the results indicated that it fell within the "Accepted" category. These data indicate that sterubin possesses all drug-likeness properties, as confirmed by ADMET analysis, as shown in Table 1. The Binding DB database was examined for potential sterubin gene targets. These showed that 58 target genes were associated with sterubin Table 2. Additional studies have been conducted using these target genes. The 58 target genes of sterubin were submitted to the STRING database with the Homo sapiens filter as a species to construct a protein-protein interaction network. The PPI network nodes and related interactions revealed how various targets interact with multiple targets during disease development. To visualize the results, the findings were loaded into Cytoscape (Figure 2). The size and color of the circles vary depending on the degree value. The PPI network comprised 58 nodes and 83 edges. According to the Cytoscape Network Analyzer, the top 10 targets were selected as the core targets, as shown in (Figure 3). These might be the main sterubin targets that support the pharmacological activity of the compound. GO enrichment analysis with the aid of the DAVID tool was employed to gain further insights into the 48 genes that were identified. The top 10 significantly enriched items in the BP, MF, and CC categories were chosen based on P<0.05, as shown in (Figure 3). The Benjamini-Hochberg process was employed to correct the p-values. BP (117 records), MF (51records) and CC (22 records) respectively. Bubble plots of bioprocesses and pathways were drawn by uploading the data to the bioinformatics platform (Figure 4). Target proteins in the BP category were mainly involved in signal transduction, positive and negative regulation of transcription from the RNA polymerase II promoter, responses to drugs and xenobiotic stimuli, negative regulation of gene expression, positive regulation of gene expression, and negative regulation of the apoptotic process. Protein binding, identical protein binding, zinc ion binding, protein homo-dimerization activity, enzyme binding, DNA binding, and other main MF categories are only a few examples. The plasma membrane, cytosol, nucleoplasm, extracellular exosome, extracellular area, macromolecular complex, mitochondrion, and cell surface were among the target proteins in CC.

**Figure 5:**
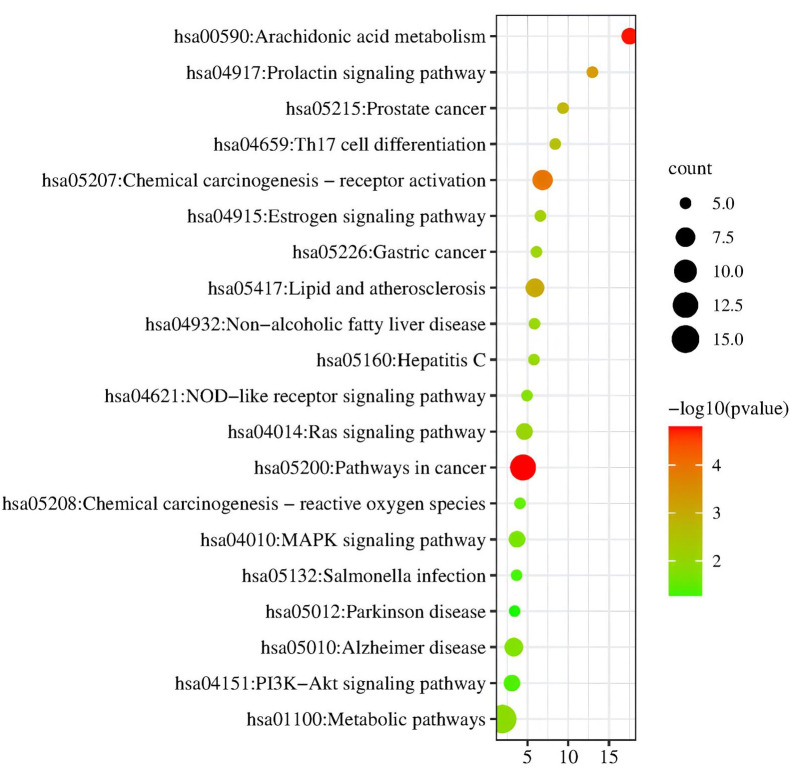
KEGG Enrichment analysis of target gene.

**Figure 6:**
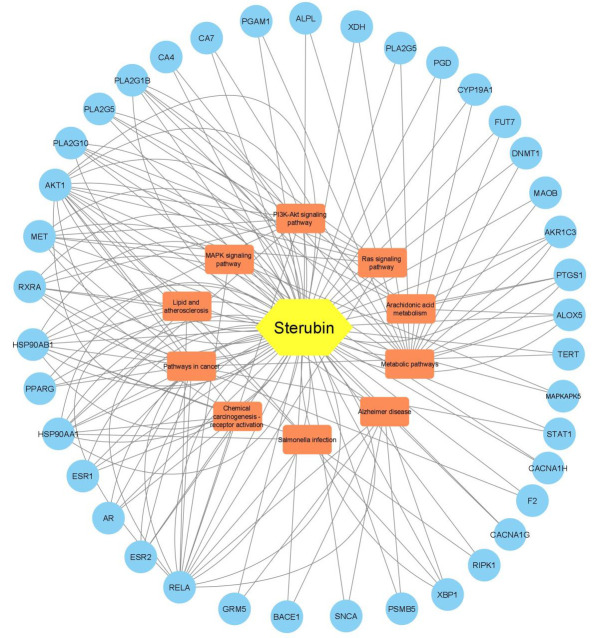
Sterubin-target-pathway network

Using the DAVID tool, we also performed KEGG enrichment analysis on these potential genes. KEGG pathway enrichment analysis identified 32 probable target genes from 48 target genes, and 10 signal pathways were strongly associated with the target genes (P<0.05). Figure 5 shows 20 pathways and their enrichment ratios. According to KEGG pathway analysis, the metabolic pathways (hsa01100), cancer-related pathways (hsa05200), chemical carcinogenesis-receptor activation (hsa05207), Alzheimer's disease (hsa05010), lipid and atherosclerosis (hsa05417), PI3K-Akt signalling (hsa04151), MAPK signalling pathway (hsa04010), Ras signalling pathway (hsa04014), arachidonic acid metabolism (hsa00590), and salmonella infection (hsa05132) were among the pathways that were significantly enriched. Using Cytoscape 3.7.2, we created a drug-target-pathway network diagram to more clearly show how sterubin, targets, and pathway interact. Figure 6 depicts a network with 49 nodes and 59 edges. The compound was represented using a yellow hexagonal; targets were represented using blue circle, and pathways using brown-square. The relationship between receptor-ligand interactions and pharmacodynamics pathways is facilitated by signalling pathways, which are a crucial component of systemic pharmacology. A target-pathway signalling network was created by placing all target proteins that interacts sterubin in the top 10 KEGG pathways.

Molecular docking was conducted with the top ten target genes, namely HSP90 AA 1, AKT-1, ESR-1, RELA, ESR-2, AR, APP, PPAR-δ, STAT1, and HSP90 AB 1, which were carefully chosen through a systematic examination of the PPI network. Table 3 shows the target PDB ID, resolution, active site, and target specification criteria. Data on the top ten docked results of sterubin with selected targets are provided in the supplemental Table 4. The results of the top five affinities, ranked from the smallest to the largest, and visualized using Discovery Studio software. The binding affinity of sterubin to APP was the lowest, at -8.83 Kcal/mol. And rest of the target (AR, HSP90 AB1, ESR-2, AKT-1, ESR-1, PPAR-δ, HSP90 AA 1 and RELA) dock scores were -8.33 Kcal/mol, -8.23 Kcal/mol, -8.17 Kcal/mol, -8.10 Kcal/mol, -7.43 Kcal/mol, -7.15 Kcal/mol, -6.54 Kcal/mol, and -5.94 Kcal/mol. According to a docking study, sterubin has significant-to-moderate interactions with these targets. Sterubin is a promising lead molecule for combating Alzheimer's disease and has a better score (APP -8.59 kJ/mol) than the rest of the targets.

Previous studies [13-14,15] have shown that sterubin exhibits superior neuro-protective, anti-inflammatory, and antioxidant activities. It was also evaluated in a rat model of chemical-induced cognitive impairment, and the results showed a significant decrease in oxidative stress and inflammatory markers, and improved behavioural studies. As a result, more preclinical studies are needed to examine the potential of sterubin compounds for treating Alzheimer's disease in preclinical studies.

Network pharmacology is a rapidly advancing field in drug development and it involves the integration of systematic medicine and information science [12]. In an effort to uncover the underlying mechanisms of synergistic therapeutic effects of traditional drugs, an in silico method was employed to construct a "protein-compound/disease-gene" network [16]. This approach has shifted the focus from a traditional "one target, one drug" model to a "network-target, multiple-component therapeutics" concept. By utilizing this network analysis technique, not only were significant biological features and genes related to sterubin identified, but also GO and KEGG enrichment analyses were conducted. This approach has the potential to expedite the drug development process by initially examining, screening, and optimizing various essential pharmacological characteristics.

## Conclusions:

It is of interest to document the network and molecular docking analysis data of sterubin with potential targets to glean insights. Hence, we document the analysis of 32 target genes and (or) its gene products and its molecular docking analysis with sterubin for further consideration in drug discovery.

## Figures and Tables

**Table 1:** ADMET analysis of sterubin

**Molecular Weight**	**Absorption**			**Distribution**		**Metabolism**		**Excretion**	**Toxicity**				
	**WS**	**IS**	**SP**	**BBB**	**CNSP**	**CYP3A4**	**CYP2C19**	**TC**	**MTD**	**ORAT**	**HT**	**SS**	**AMES**
302.282	-3.223	83.94	-2.736	-1.13	-3.112	NO	No	0.102	-0.011	2.588	No	No	No

**Table 2:** Potential genes targeted by sterubin

**S.N**	**Gene**	**UniProt ID**	**Description**
1	PGD	P52209	6-phosphogluconate dehydrogenase
2	ACHE	P22303	Acetylcholinesterase
3	AKR1C3	P42330	Aldo-keto reductase family 1 member C3
4	ALPL	P05186	Alkaline phosphatase, tissue-nonspecific isozyme
5	FUT7	Q11130	Alpha-(1,3)-fucosyltransferase 7
6	SNCA	P37840	Alpha-synuclein
7	MAOB	P27338	Amine oxidase [flavin-containing] B
8	APP	P05067	Amyloid-beta precursor protein
9	AR	P10275	Androgen receptor
10	Bcl-2	P10415	Apoptosis regulator Bcl-2
11	CYP19A1	P11511	Aromatase
12	ABCB1	P08183	ATP-dependent translocase ABCB1
13	BACE1	P56817	Beta-secretase 1
14	CA1	P00915	Carbonic anhydrase 1
15	CA12	O43570	Carbonic anhydrase 12
16	CA2	P00918	Carbonic anhydrase 2
17	CA4	P22748	Carbonic anhydrase 4
18	CA7	P43166	Carbonic anhydrase 7
19	CTSV	O60911	Cathepsin L2
20	CYP4A11	Q02928	Cytochrome P450 1B1
21	DRD2	P14416	D (2) dopamine receptor
22	DPP4	P27487	Dipeptidyl peptidase 4
23	DNMT1	P26358	DNA (cytosine-5)-methyltransferase 1
24	RAD52	P43351	DNA repair protein RAD52 homolog
25	DYRK1A	Q13627	Dual specificity tyrosine-phosphorylation-regulated kinase 1A
26	ELAVL3	Q14576	ELAV-like protein 3
27	ESR1	P03372	Estrogen receptor
28	ESR2	Q92731	Estrogen receptor beta
29	PLA2G10	O15496	Group 10 secretory phospholipase A2
30	HSP90AA1	P07900	Heat shock protein HSP 90-alpha
31	HSP90AB1	P08238	Heat shock protein HSP 90-beta
32	MET	P08581	Hepatocyte growth factor receptor
33	HRH3	Q9Y5N1	Histamine H3 receptor
34	MAPKAPK5	Q8IW41	MAP kinase-activated protein kinase 5
35	GRM5	P41594	Metabotropic glutamate receptor 5
36	PPARG	P37231	Peroxisome proliferator-activated receptor gamma
37	PGAM1	P18669	Phosphoglycerate mutase 1
38	PLA2G5	P39877	Phospholipase A2 group V
39	PLA2G1B	P04054	Phospholipase A2, membrane associated
40	SERPINE1	P05121	Plasminogen activator inhibitor 1
41	ALOX5	P09917	Polyunsaturated fatty acid 5-lipoxygenase
42	PTGS1	P23219	Prostaglandin G/H synthase 1
43	PSMB5	P28074	Proteasome subunit beta type-5
44	F2	P00734	Prothrombin
45	AKT1	P31749	RAC-alpha serine/threonine-protein kinase
46	RIPK1	Q13546	Receptor-interacting serine/threonine-protein kinase 1
47	RXRA	P19793	Retinoic acid receptor RXR-alpha
48	SHBG	P04278	Sex hormone-binding globulin
49	STAT1	P42224	Signal transducer and activator of transcription 1-alpha/beta
50	SLCO2B1	O94956	Solute carrier organic anion transporter family member 2B1
51	TERT	O14746	Telomerase reverse transcriptase
52	RELA	Q04206	Transcription factor p65
53	TTR	P02766	Transthyretin
54	PTPN1	P18031	Tyrosine-protein phosphatase non-receptor type 1
55	CACNA1G	O43497	Voltage-dependent T-type calcium channel subunit alpha-1G
56	CACNA1H	O95180	Voltage-dependent T-type calcium channel subunit alpha-1H
57	XBP1	P17861	X-box-binding protein 1
58	XDH	P47989	Xanthine dehydrogenase/oxidase

**Table 3:** Protein speciations

**S.NO**	**PROTEIN**	**PBD CODE**	**RESOLUTION(Å)**	**ORGANISM**	**METHOD**	**Active site**
1	HSP90 AA 1	2YKJ	1.46Å	Homo Sapiens	X-ray diffraction	Leu 103, Try 139
2	AKT-1	6HHH	2.7Å	Homo Sapiens	X-ray diffraction	Glu 85 a
3	ESR-1	3ERT	1.9Å	Homo Sapiens	X-ray diffraction	Glu 353, Arg 394
4	RELA/NFKB p65	7BIW	1.2Å	Homo Sapiens	X-ray diffraction	Arg 56, Arg 139, Tyr 130, Asn 175, Asp 215
5	ESR-2	1X78	2.3Å	Homo Sapiens	X-ray diffraction	Leu 610, Thr 611, Thr 612
6	AR	3L3X	1.55Å	Homo Sapiens	X-ray diffraction	Asn 705, Arg 752, Thr 877
7	APP	3PMR	2.11Å	Homo Sapiens	X-ray diffraction	Lys 484, Arg 491
8	PPAR-δ	8DSZ	2.5Å	Homo Sapiens	X-ray diffraction	Ser 317, Tyr 355
9	STAT1	1yvl	3Å	Homo Sapiens	X-ray diffraction	Lys 584, Ser 606, Ser 604, Arg 602, Ala 630, His 629, Glu 632, Tyr 651
10	HSP90 AB 1	3NMQ	2.2Å	Homo Sapiens	X-ray diffraction	Asp 93

**Table 4:** Sterubin-target molecular docking analysis

**Bioactive Component**	**Target**	**PDB ID**	**Binding energy (Kcal/mol)**	**Description**
Sterubin	HSP90 AA 1	2YKJ	-6.54	Heat shock protein 90 alpha family class A Member 1
	AKT-1	6HHH	-8.1	AKT serine/threonine kinase 1
	ESR-1	3ERT	-7.43	Estrogen receptor
	RELA	7BIW	-5.94	Transcription factor p65
	ESR-2	1X78	-8.17	Estrogen receptor beta
	AR	3L3X	-8.33	Androgen receptor
	APP	3PMR	-8.79	Amyloid-beta precursor protein
	PPAR-δ	8DSZ	-7.15	Peroxisome proliferator-activated receptor gamma
	STAT1	1yvl	-5.37	Signal transducer and activator of transcription 1-alpha/beta
	HSP90 AB 1	3NMQ	-8.23	Heat shock protein 90 alpha family class B Member 1
